# Protocol for olefin metathesis reactions of hydrophobic substrates performed in aqueous emulsion with mechanical stirring or with microwaves support

**DOI:** 10.1016/j.xpro.2022.101671

**Published:** 2022-09-21

**Authors:** Agata Tyszka-Gumkowska, Anna Kajetanowicz, Karol Grela

**Affiliations:** 1 Biological and Chemical Research Centre, Faculty of Chemistry, University of Warsaw, Żwirki i Wigury 101, 02-089 Warsaw, Poland

**Keywords:** Chemistry, Environmental sciences

## Abstract

In the presented protocol, we describe the olefin metathesis of hydrophobic substrates in water emulsions using ruthenium catalysts in the presence of air. We detail the testing of mechanical foaming for emulsification and the use of microwave heating to optimize metathesis reaction efficiency. By utilizing relatively low catalyst loading and ensuring simple product isolation, the steps outlined in this protocol extend known methods for the aqueous metathesis techniques.

For complete details on the use and execution of this protocol, please refer to Tyszka-Gumkowska et al. (2022).

## Highlights

• Synthesis and purification of 9-chloro-2-methylnon-4-ene and 11-hydroxyundec-2-enal
• Application of water as a sustainable diluent for the metathesis reaction in air
• Utilization of microwaves for boosting metathesis under aqueous conditions
• Protocol with low catalyst loading and simple product isolation

## Abstract (editor highlights)

Publisher’s note: Undertaking any experimental protocol requires adherence to local institutional guidelines for laboratory safety and ethics.

## Graphical abstract

**Figure:**
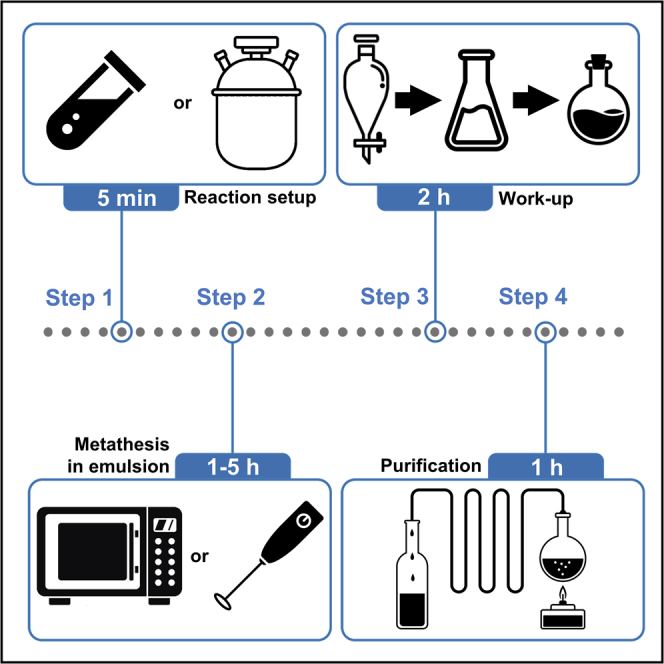

## Before you begin

Olefin metathesis reaction has become one of the most convenient methods for the construction of carbon-carbon double bonds. Mostly due to the discovery of robust and active ruthenium catalysts this transformation has found wide applications in academia and industry (Grela, 2014; Ogba et al., 2018). As in other organometallic transformations, metathesis reactions usually require carefully dried and degassed aromatic or chlorinated solvents to prevent catalyst decomposition and side products formation. Unfortunately, the described conditions are far from the ideal Green Chemistry protocols, which is a serious limitation to the even more ubiquitous application of metathesis reactions (Clavier et al., 2007). To address this issue, scientific community attempt several trials to perform this reaction under aqueous conditions, since water represents the major green solvent: cheap, easily available, and non-toxic.

The most straightforward strategy to achieve this goal was to perform reactions ’on water‘ in a heterogenous system with water insoluble metathesis catalysts, taking advantage of the hydrophobic effect that accelerates the reaction rate (Davis and Sinou, 2002). On the other hand, the addition of various amphiphilic substances (surfactants) allowed for effective olefin metathesis ’in water‘. These additives lead to the formation of micelles capable of accommodating water-insoluble substrate(s) along with a metathesis catalyst and ensure an effective course of the metathesis reaction (Lipshutz et al., 2008; Lipshutz and Ghorai, 2010).

A similar strategy involved specially designed *catsurfs* (catalyst+surfactant) – catalysts able to acting simultaneously as initiators and surfactants (Gawin et al., 2010). Recently, several water-soluble catalysts have also been presented, gained by incorporation of ionic tags or PEGs into classical catalysts structure (Hong and Grubbs, 2006; Olszewski et al., 2020; Skowerski et al., 2012; Wang et al., 2015). Nevertheless, relatively high loading of these tailor-made catalysts is usually required.

Instead of using surfactants and specialized catalysts for olefin metathesis, a similar performance can be obtained by ultrasonication of a lipophilic substrate and a commercially available hydrophobic catalyst in an emulsion system (Gułajski et al., 2008, 2019). Using this protocol, the hydrophobic catalyst and substrates are encapsulated in small droplets of reaction media, allowing the metathesis reaction to proceed smoothly.

Herein, we present our attempt to perform olefin metathesis reactions under aqueous conditions, stressing out the practical aspects of this process performed on a larger scale, when one needs to consider not only the chemical but also the economical parameters of the transformation. In this respect, it seems reasonable to us to use water as a diluent to suspend the reactants, ensuring convenient stirring and heat transfer. To probe the usefulness of the developed conditions on the manufacturing of fine chemicals, we decided to study lipophilic liquid substrates in olefin metathesis using water as a reaction medium. These conditions offer a convenient tool for conducting olefin metathesis under more environmentally and user-friendly conditions (Tyszka-Gumkowska et al., 2022).

### Preparation of FixCatBARF catalyst

**Timing: 3 h**

**Scheme 1:**
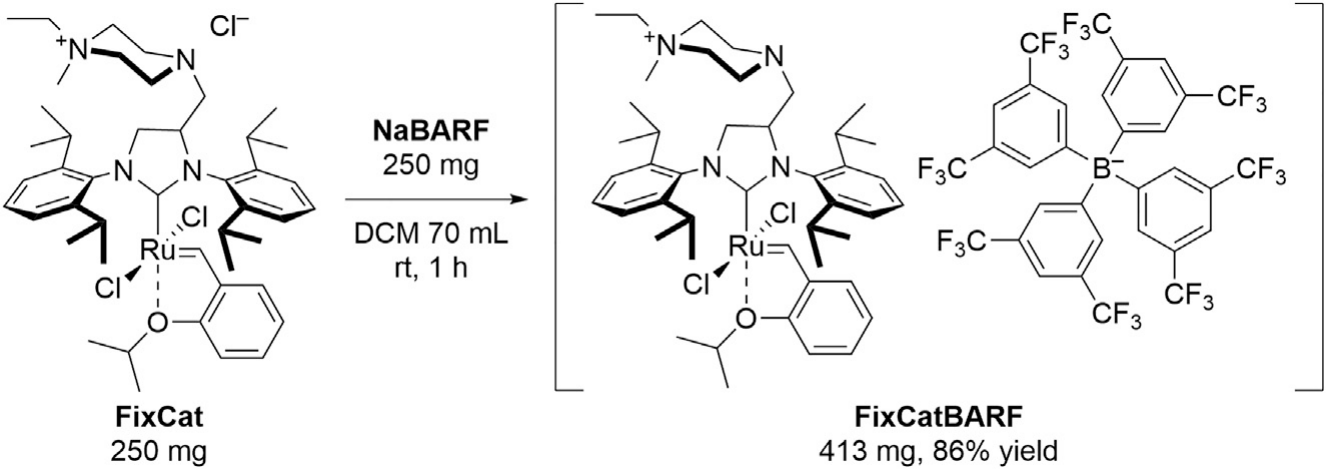
Synthesis of FixCatBARF catalyst

These steps describe preparation of FixCatBARF catalyst, by ion exchange reaction between NaBARF and FixCat catalyst (Scheme 1). Reaction should be performed using Schlenk line equipment consisting of two neck round bottom flask or Schlenk flask with a vacuum manifold which is connected to a vacuum pump, and an inert gas manifold which is connected to a source of commercially available and high quality argon (HiQ Argon 6.0). All reaction steps should be carried out in a well ventilated fume hood.

1. Evacuate the oven-dried Schlenk flask (volume 100 mL) for at least 15 min and backfill it with argon (flow rate ∼4 L/min, pressure 0.2 bar).
2. Under argon atmosphere add NaBARF (250 mg, 0.282 mmol) to the Schlenk flask, carefully evacuate and backfill the flask with argon three-times more.
3. Under argon atmosphere add 70 mL of anhydrous DCM.

***Note:*** NaBARF will not be fully dissolved in DCM.

4. After 15 min of stirring add commercially available FixCat catalysts in one portion under argon atmosphere (1.0 equiv., 250 mg, 0.282 mmol) and stir for 1 h at ∼20°C–25°C.

***Note:*** The catalyst can be weighed out in air, but should be stored under argon in the refrigerator at approximately 4°C.

5. Concentrate the mixture on rotary evaporator (35°C, 400 mbar, ∼20 min).
6. Place the residue on a pad with neutral aluminum oxide Brockmann activity IV (∼15 cm length, 3 cm diameter), wash with DCM, and collect highly movable green band.
7. Evaporate solution of the product and dry it under high vacuum (∼3·10^−2^ mbar, at ∼20°C–25°C for 16 h) to obtain FixCatBARF as a green powder (413 mg, 0.241 mmol, 86%).

^1^H NMR (400 MHz, CD_2_Cl_2_) δ 16.12 (s, 1H), 7.73 (s, 8H), 7.65 (t, *J* = 7.6 Hz, 1H), 7.57 (s, 4H), 7.56 – 7.49 (m, 2H), 7.45 (d, *J* = 7.7 Hz, 2H), 7.42 – 7.33 (m, 2H), 6.93 – 6.78 (m, 3H), 5.01 – 4.84 (m, 1H), 4.48 – 4.27 (m, 2H), 4.22 – 4.02 (m, 1H), 3.84 – 3.67 (m, 1H), 3.63 – 3.53 (m, 1H), 3.53 – 3.36 (m, 1H), 3.32 – 3.25 (m, 6H), 2.93 (s, 3H), 2.84 – 2.76 (m, 3H), 2.69 – 2.59 (m, 1H), 1.57 – 1.53 (m, 8H), 1.47 – 1.43 (m, 2H), 1.36 – 1.22 (m, 22H), 1.13 – 1.07 (m, 2H), 0.89 – 0.83 (m, 2H). ^13^C NMR (101 MHz, CD_2_Cl_2_) δ 289.5, 217.6, 162.1 (q, ^1^*J*_*C-B*_ = 49.8 Hz), 152.7, 149.5, 144.0, 137.0, 135.2, 130.5, 130.4, 130.1, 129.2 (q, ^2^*J*_*C-F*_ = 31.7 Hz), 126.0, 125.7, 125.0 (q, ^1^*J*_*C-F*_ = 272.5 Hz), 124.5, 122.9, 122.7, 117.9, 113.4, 75.7, 63.4, 60.8, 60.1, 53.8, 47.6, 46.8, 29.3, 28.2, 25.7, 24.3, 23.2, 22.0, 7.7. ^19^F NMR (376 MHz, CD_2_Cl_2_) δ −62.8. ^11^B NMR (128 MHz, CD_2_Cl_2_) δ −6.6. HRMS ESI: positive (*m*/*z*) calc. for C_45_H_67_Cl_2_N_4_ORu^+^ [M]^+^ 851.3730, found 851.3727; negative (*m*/*z*) calc. for C_32_H_12_BF_24_^−^ [M]^−^ 863.0643, found 863.0662.

**CRITICAL:** NaBARF should be a high quality, anhydrous, and white loose powder. The compound should be stored under an argon atmosphere in the refrigerator at approximately 4°C.

## Key resources table

**Table:** 

REAGENT or RESOURCE	SOURCE	IDENTIFIER
**Chemicals, peptides, and recombinant proteins**		

**UltraCat** (1-(2,6-diethylphenyl)-3,5,5-trimethyl-3-phenylpyrrolidin-2-ylidene)dichloro(2-isopropoxy-5-nitrobenzylidene)ruthenium(II)	Apeiron Synthesis	Cat#AS2091 CAS: 2106819-64-9
**FixCat** (1,3-bis(2,6-diisopropylphenyl)-4-((4-ethyl-4-methylpiperazin-1-ium-1-yl)methyl)imidazolidin-2-ylidene)dichloro(2-isopropoxybenzylidene)ruthenium(II) chloride	Apeiron Synthesis	Cat#AS2061 CAS: 1799947-97-9
**FixCatBARF** (1,3-bis(2,6-diisopropylphenyl)-4-((4-ethyl-4-methylpiperazin-1-ium-1-yl)methyl)imidazolidin-2-ylidene)dichloro(2-isopropoxybenzylidene)ruthenium(II) tetrakis[3,5-bis(trifluoromethyl)phenyl]borate	Synthetized in our lab	this work
SnatchCat	Apeiron Synthesis	Cat#AS1033 CAS: 51641-96-4
NaBARF	Apollo Scientific	Cat#PC1999 CAS: 79060-88-1
6-chlorohexene	Alfa Aesar	Cat#H53396.14 CAS: 928-89-2
4-methyl-1-pentene	Sigma-Aldrich	Cat#M67400 CAS: 691-37-2
crotonaldehyde	Sigma-Aldrich	Cat#262668 CAS: 123-73-9
9-decen-1-ol	Sigma-Aldrich	Cat# 118354 CAS: 13019-22-2
anhydrous DCM	Sigma-Aldrich	Cat#270997 CAS: 75-09-2
distilled water	Linegal Chemicals	sklep.linegal.pl
ethyl acetate	Sigma-Aldrich	Cat#34858
hexene	Linegal Chemicals	sklep.linegal.pl

**Other**		

rotary evaporator Rotavapor R-100	BUCHI	www.buchi.com
silica gel (60, particle size 0.043–0.063 nm)	Merck Millipore	https://merckmillipore.com
aluminum oxide	Sigma-Aldrich	Cat#199966 CAS: 1344-28-1
milk frother	Tchibo	www.tchibo.pl
microwave reactor CEM Discover SP	CEM	www.cem.com
reaction vessels for microwave reactor	CEM	www.cem.com
five-neck glass reactor (volume 100 mL)	Quickfit	pl.vwr.com
glass vials	Linegal Chemicals	sklep.linegal.pl
measuring cylinders	VWR Collection	pl.vwr.com
round bottom flasks	VWR Collection	pl.vwr.com
separatory funnels	VWR Collection	pl.vwr.com
Erlenmeyer flasks	VWR Collection	pl.vwr.com
beakers	VWR Collection	pl.vwr.com
funnels	VWR Collection	pl.vwr.com
long funnel with a frit	VWR Collection	pl.vwr.com
PP/PE syringes	B. Braun	www.bbraun.com
single-use SS/PE needles	B. Braun	www.bbraun.com
spatulas	VWR Collection	pl.vwr.com
stirring elements	VWR Collection	pl.vwr.com
Kugel Rohr B-585 distillation apparatus with glass bulbs	BUCHI	www.buchi.com

## Materials and equipment

**Table:** 

Reagents for the synthesis of *9-chloro-2-methylnon-4-ene*		
Reagent	Final concentration [mmol/mL_AcOEt_]	Amount
6-chlorohexene	4.12	6.55 mL
4-methyl-1-pentene	12.34	18.80 mL
UltraCat	0.0412	801 mg
distilled water	N/A	45 mL
ethyl acetate	N/A	12 mL
SnatchCat	N/A	474 mg

Reagents can be stored at ∼4°C temperature for ∼12 months

**Table:** 

Reagents for the synthesis of *11-hydroxyundec-2-enal*		
Reagent	Final concentration	Amount
9-decen-1-ol	N/A	2.22 mL
crotonaldehyde	N/A	3.94 mL
FixCatBARF	N/A	206 mg
distilled water	N/A	24 mL
SnatchCat	N/A	119 mg

Reagents can be stored at ∼4°C temperature for ∼12 months

**CRITICAL:** Ruthenium complexes are potentially mutagenic and toxic compounds. 9-decen-1-ol, crotonaldehyde, and 6-chlorohexene are flammable, hazards for environment and irritating reagents, when 4-methyl-1-pentene is also highly volatile. Avoid contact of these substances with skin and eyes, and do not inhale or ingest them. Use personal protective equipment such as lab coat, goggles and gloves. All reaction setups should be carried out in well ventilated fume hood.

***Alternatives:*** Reagents can be replaced by those obtained from other suppliers, as long as they are of the same quality and purity as those used in presented protocols; except five-neck glass reactor for synthesis of *9-chloro-2-methylnon-4-ene* round bottom flask closed with septum can be used; milk frother can be replaced with mechanical stirrer or strong magnetic stirrer; Kugel Rohr distillation apparatus can be replaced with classical glassware for distillation under reduce pressure; CEM Discover SP for synthesis of *11-hydroxyundec-2-enal* can be replace by other microwave reactor allowing for temperature and power control; long funnel with a frit can be replace by chromatography column glassware.

The resources listed in the below table were based on our experience. Generally, the chemicals and resources can be purchased from any reliable commercial sources and do not need to be limited to those listed in our table.

## Step-by-step method details

### Part 1: Synthesis of 9-chloro-2-methylnon-4-ene

**Timing: 5.5 h**

**Scheme 2:**
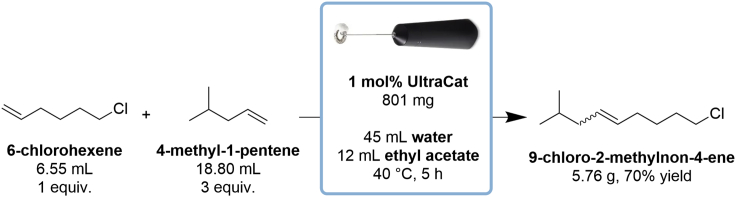
Synthesis of 9-chloro-2-methylnon-4-ene by metathesis reaction in emulsion system using milk frother

These steps describe the synthesis of *9-chloro-2-methylnon-4-ene* by the metathesis reaction of 6-chlorohexene and 4-methyl-1-pentene using UltraCat as a catalyst (Scheme 2). Moreover, in this protocol, milk frother is used for emulsification of the reaction mixture: water, ethyl acetate, lipophilic substrates, and catalyst. All reaction steps should be carried out in well ventilated fume hood.

***Note:*** Synthesis of title compound *9-chloro-2-methylnon-4-ene* can be alternatively performed under classical conditions using 4-methyltetrahydropyran as a solvent, and 88% NMR yield was observed by authors (Nienałtowski et al., 2020).

**Figure 1:**
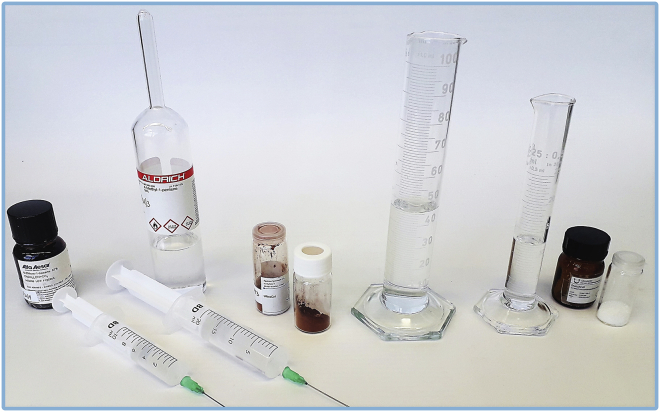
Reagents prepared for the synthesis of *9-chloro-2-methylnon-4-ene*

***Note:*** The catalyst can be weighed out in air, but it should be stored under argon in the refrigerator at approximately 4°C. In these studies, it was possible for us to use a lower quality UltraCat catalyst (60% purity) as it gave identical result as the freshly purified one.

**Figure 2:**
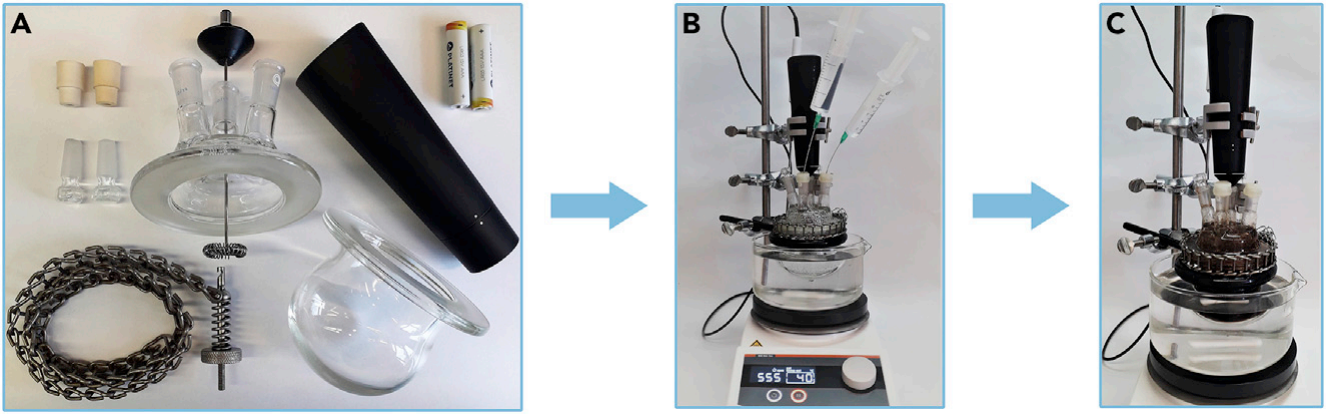
Reaction setup (A–C) (A) equipment and glassware (B) addition of substrates (C) reaction mixture at the end of metathesis reaction.

1. Preparation of substrates and solvents (Figure 1):
  a. measure out substrates with a PP/PE syringes tipped with a single-use SS/PE needles 0.80 × 120 mm: 6.55 mL of 6-chlorohexene and 18.80 mL of 4-methyl-1-pentene;
  b. weigh out 801 mg of UltraCat catalyst in the glass vial.
  c. measure out 45 mL of distilled water and 12 mL of ethyl acetate with the cylinder;
  d. weigh out 474 mg of SnatchCat scavenger in the glass vial.

2. Set up the reaction (Figure 2):
  a. prepare the reactor (volume 100 mL) by placing the tip of the milk frother in one of the necks;
  b. connect the upper and lower parts of the glass reactor: lubricate the ground glass part with hydrocarbon-based grease and tighten them with a chain clamp;
  c. pour water into the reactor through one of the necks with the help of a funnel and heat it to 40°C;
  d. after reaching the appropriate temperature, inject into the reactor the 6-chlorohexene and 4-methyl-1-pentene prepared in syringes through the neck of the reactor ended with a rubber septum;
  e. turn on the milk frother to form an emulsion;
  f. once the reaction has reached the desired temperature (40°C), add the UltraCat catalyst and pour ethyl acetate into the reactor; troubleshooting 1;
  g. continue stirring for 5 h at 40°C.

**Pause point:** Once the temperature and stirring mixer have stabilized, the reaction is ready to run.

***Optional:*** Presented five-neck glass reactor can be replace by round bottom flask (volume 100 mL) with septum. Except milk frother some mechanical or magnetic stirrer can be used.

### Part 2: Purification of 9-chloro-2-methylnon-4-ene

**Timing: 2 h**

These steps describe the purification of the reaction mixture by removing the catalyst and distilling the crude product. All these steps should be carried out in well ventilated fume hood.

**Figure 3:**
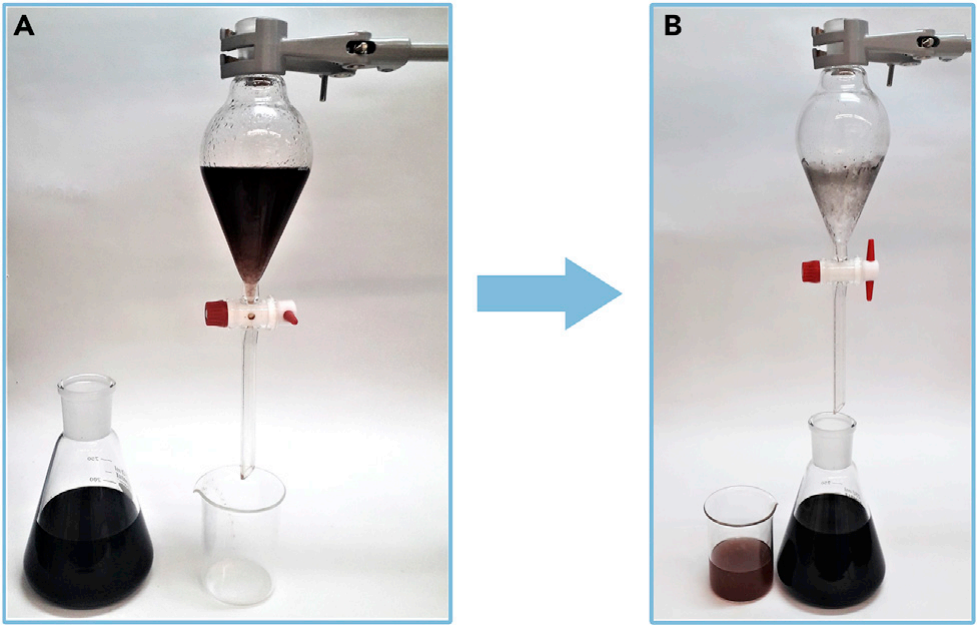
Extraction of the product from water (A and B) (A) water and organic layer mixed in the separatory funnel (B) layers separated after extraction.

***Note:*** Repeat this step two more times or until all product will be transfer to organic phase. Transfer of the product to organic layer can be monitored using thin layer chromatography (silica gel, hexane:ethyl acetate, 9:1).

3. After completion of the reaction, transfer the mixture to a 250 mL separatory funnel and separate the aqueous phase (bottom layer) from the organic one (top layer). Troubleshooting 2 and 3.
4. Place aqueous phase in beaker.
5. Transfer the organic phase to an Erlenmeyer flask.
6. Put the aqueous phase back into the separatory funnel, add 10 mL of ethyl acetate, shake the separatory funnel vigorously and, after separating the two phases, transfer organic phase to suitable Erlenmeyer flasks (Figure 3).

7. Discard the aqueous phase in the appropriate waste container.
8. Add 474 mg of SnatchCat scavenger to the organic fractions collected in the Erlenmeyer flask and stir this mixture for at least 30 min.
9. Add drying agent (MgSO_4_ or Na_2_SO_4_), gently stir it and filter into a round bottom flask. Troubleshooting 4.
10. Remove ∼95% of the solvent using rotary evaporator (35°C, 130 mbar, ∼15 min).
11. Prepare a silica gel pad (∼20 cm length, 3 cm diameter) and place the crude product on top, washing the round bottom flask with a small amount of hexane (Figure 4).

**Figure 4:**
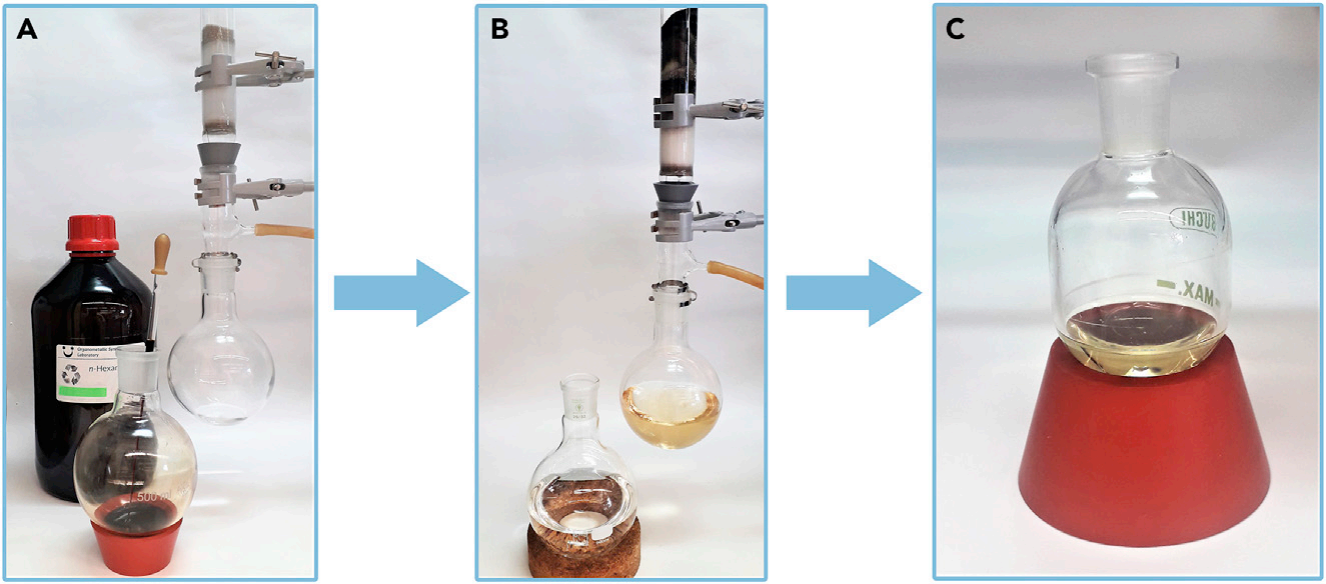
Removing catalyst residues from the reaction mixture (A–C) (A) filtration setup (B) washing the silica gel pad with hexane (C) crude product ready for the distillation.
12. Carefully wash the product off the silica gel pad with hexane under vacuum filtration.

**CRITICAL:** The dark brown residue of the catalyst after filtration should remain on top of the silica gel pad. The operation should be repeated if the catalyst residues penetrate too much into the silica gel layer and color the crude product solution. Any catalyst residues left in the crude product can cause its isomerization or decomposition.

13. Remove the solvent on a rotary evaporator (35°C, 130 mbar, ∼20 min).
14. Place the crude product in a small round bottom flask.

***Note:*** Do not use a high vacuum pump for the long-term drying of the crude product due to its volatility.

**Figure 5:**
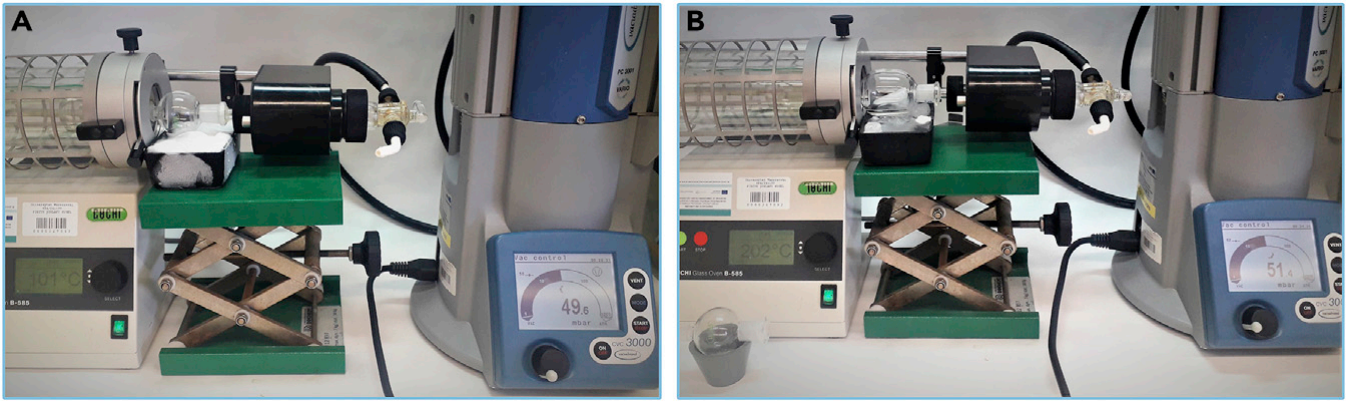
Distillation of the crude product using Kugel Rohr apparatus (A and B) (A) beginning of the process (B) distillation of actual product.

15. Purify the crude product by distillation (Figure 5):
  a. set up the parameter on Kugel Rohr distillation apparatus to distill out the unreacted starting material: 100°C, 50 rpm and 50 mbars on vacuum pump; troubleshooting 5.
  b. collect the first fraction in the bulb;
  c. place a new bulb in the apparatus, raise the temperature to 160°C, and collect another fraction;
  d. place new bulb in the apparatus, raise the temperature to 200°C, and collect the pure product;
  e. leave a higher boiling yellow residue in the round bottom flask.

***Optional:*** Kugel Rohr apparatus can be replaced by standard laboratory glassware for distillation under vacuum.

**Figure 6:**
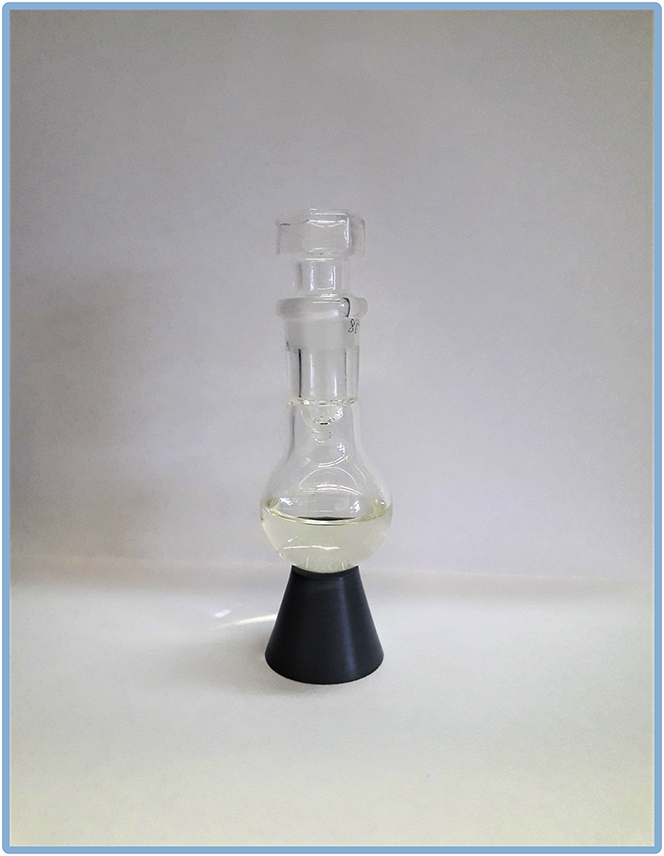
Pure 9-chloro-2-methylnon-4-ene

16. Transfer the distilled product to the fresh round bottom flask (previously tared or weighed), wash with a small amount of hexane, remove the solvent on rotary evaporator, and weight out the amount of the final compound (Figure 6).

### Part 3: Synthesis of 11-hydroxyundec-2-enal

**Timing: 1.5 h**

**Scheme 3:**
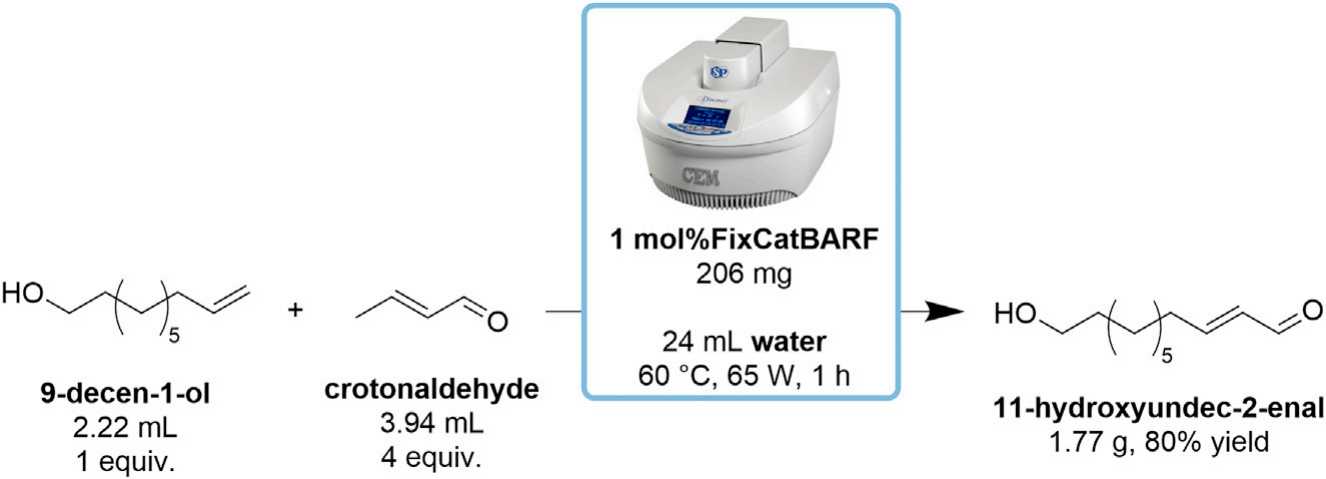
Synthesis of 11-hydroxyundec-2-enal in a microwave reactor using water as a diluent

These steps describe the synthesis of *11-hydroxyundec-2-enal* by cross-metathesis of 9-decen-1-ol and crotonaldehyde using FixCatBARF as a catalyst in a microwave reactor (Scheme 3). Reaction setup should be carried out in well ventilated fume hood.

***Note:*** Synthesis of title compound 11-hydroxyundec-2-enal can be alternatively performed in water emulsion system with ultrasounds assistance (Tyszka-Gumkowska et al., 2022) or under classical conditions in dichloromethane (Aubineau and Cossy, 2018), nevertheless in both cases lower yield were observed by authors: 85% (NMR yield) and 65% (isolated yield), respectively.

**Figure 7:**
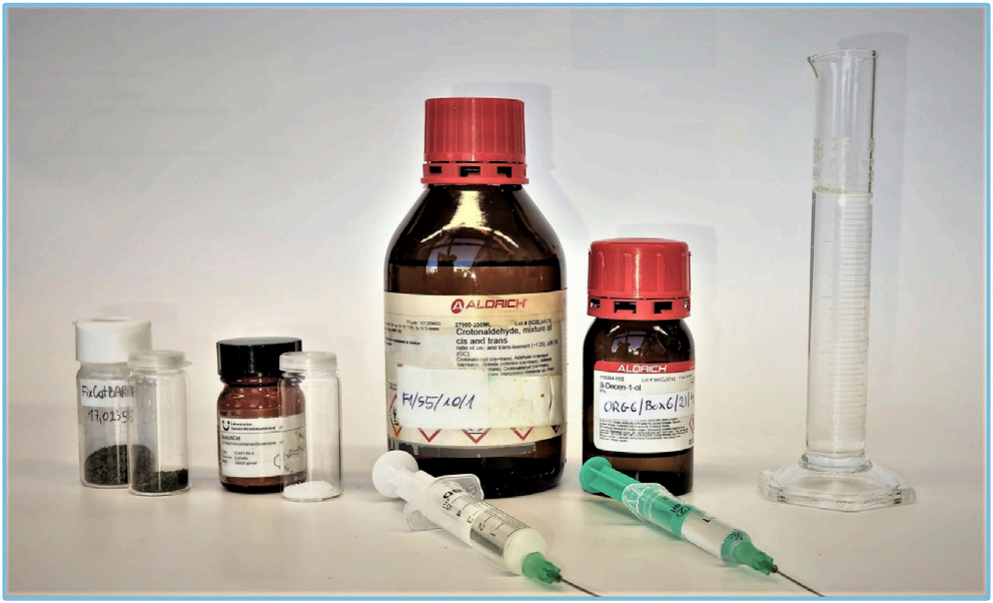
Reagents prepared for the synthesis of *11-hydroxyundec-2-enal*

***Note:*** The catalyst can be weighed out in air, but should be stored under argon in the refrigerator at approximately 4°C.

**Figure 8:**
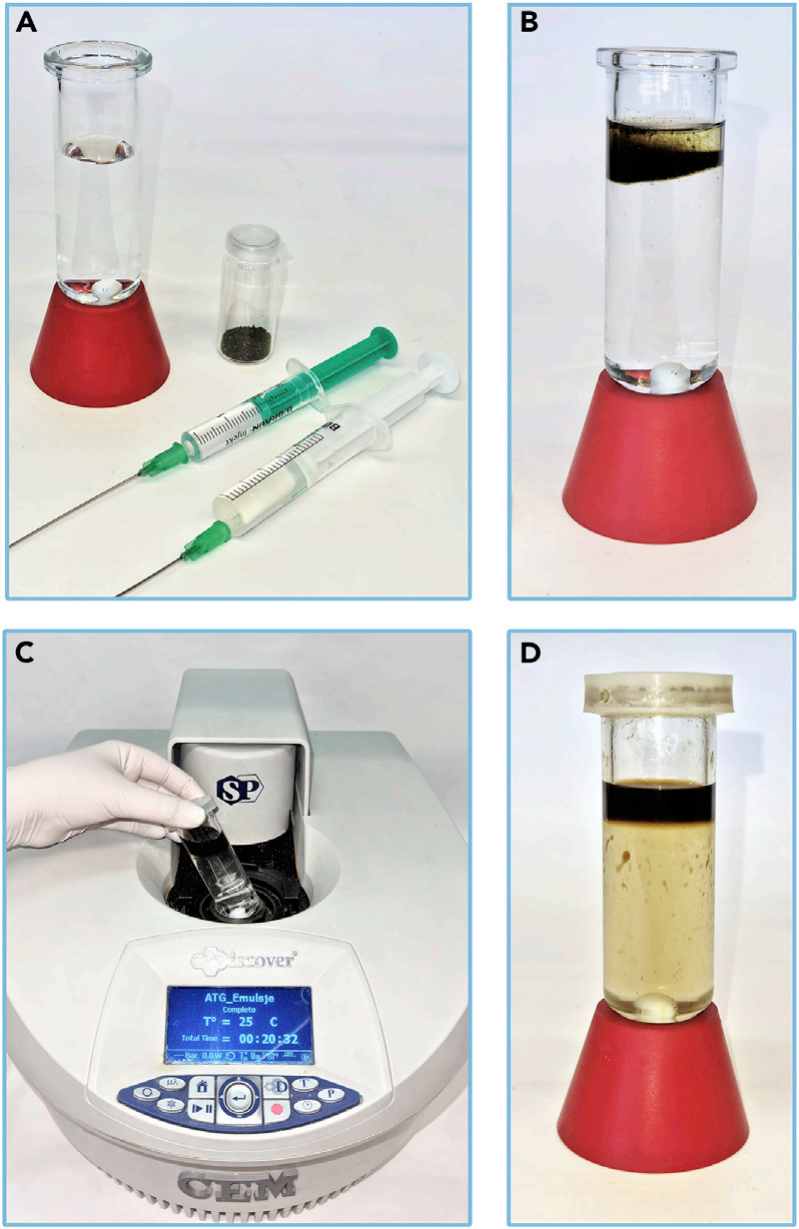
Reaction setup (A–D) (A) prepared reaction vessel and substrates (B) pre-reaction mixture (C) vessel placing in the microwave reactor (D) post-reaction mixture.

17. Preparation of reagents and solvents (Figure 7):
  a. measure out substrates with a PP/PE syringes tipped with a single-use SS/PE needles 0.80 × 120 mm: 2.22 mL of 9-decen-1-ol and 3.94 mL of crotonaldehyde;
  b. weigh out 206 mg of FixCatBARF catalyst in the glass vial;
  c. weigh out 119 mg of SnatchCat in the glass vial;
  d. measure out 24 mL of distilled water with a cylinder.

18. Set up the reaction (Figure 8):
  a. pour water in a microwave reaction vessel (volume 35 mL) with neodymium stirring element;
  b. inject there substrates: 9-decen-1-ol and crotonaldehyde;
  c. in one portion add FixCatBARF catalyst to the reaction vessel;
  d. close the vessel with cap and place into the microwave reactor;
  e. set up the reactor parameters: 65 W, 60°C, 1 h.

**CRITICAL:** Choose a fairly large stirring element, ideally neodymium one, as mixing in a microwave reactor is difficult. Efficient mixing is necessary to form sufficiently small droplets of a suspension of the substrates and the catalyst in water.

**Pause point:** Once the temperature is reached (∼2 min), the reaction will run for 1 h. The microwave reactor then cools the reaction mixture to ∼20°C–25°C (∼10 min).

### Part 4: Purification of *11-hydroxyundec-2-enal*

**Timing: 3 h**

These steps describe the purification of the reaction mixture by removing the catalyst and distilling the crude product. All these steps should be carried out in well ventilated fume hood.

**Figure 9:**
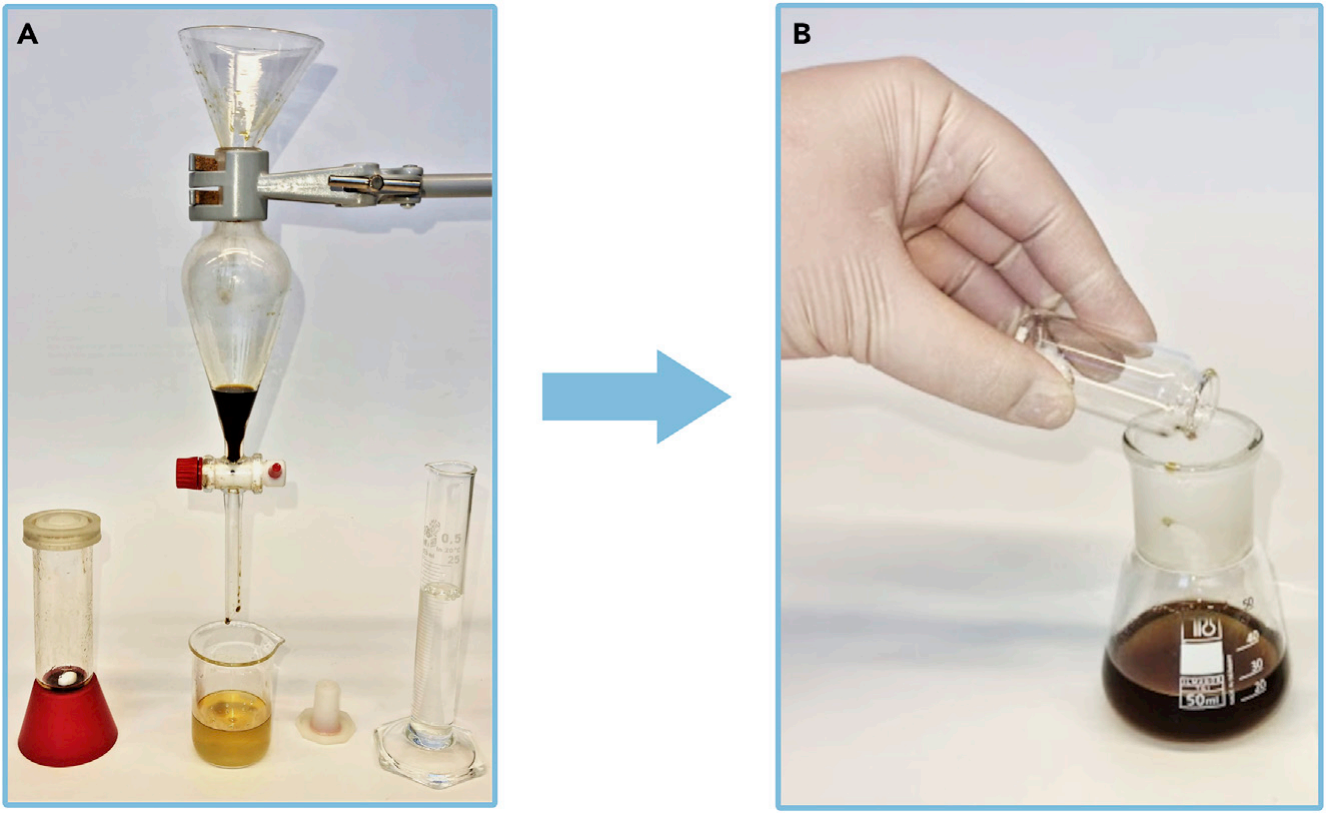
Separation of the crude product from aqueous phase (A and B) (A) extraction (B) addition of SnatchCat scavenger.

19. After completion of the reaction, transfer the mixture to a 50 mL separatory funnel and separate the aqueous phase (bottom layer) from the organic one (top layer). Troubleshooting 2 and 3.
20. Place aqueous phase in a beaker.
21. Transfer the organic phase to Erlenmeyer flask.
22. Put the aqueous phase back into the separatory funnel, add 5 mL of ethyl acetate, shake the separatory funnel vigorously and, after separating the two phases, transfer them to a suitable Erlenmeyer flasks (Figure 9).

**CRITICAL:** Compounds having an aldehyde group in aliphatic chain quite readily dissolve in water. That is why the extraction step should be repeated at least five times or until no product is present in the water phase. Transfer of the product to organic layer can be monitored using thin layer chromatography (silica gel, hexane:ethyl acetate, 9:1).

23. Discard the aqueous phase in the appropriate waste container.
24. Add 119 mg of SnatchCat scavenger to the organic phase.
25. Stir this mixture vigorously for at least 30 min.

***Note:*** The mixture of crude product and SnatchCat can be stored for 24 h in the freezer at approximately −20°C.

26. Add drying agent portion wise (MgSO_4_ or Na_2_SO_4_), gently mix it and filter into a round bottom flask. Troubleshooting 4.
27. Remove ∼95% of the solvent with a rotary evaporator (35°C, 130 mbar, ∼10 min).
28. Prepare a silica gel pad (∼10 cm length, 3 cm diameter) and place the crude product on top, washing the round bottom flask with a small amount of hexane (Figure 10).

**Figure 10:**
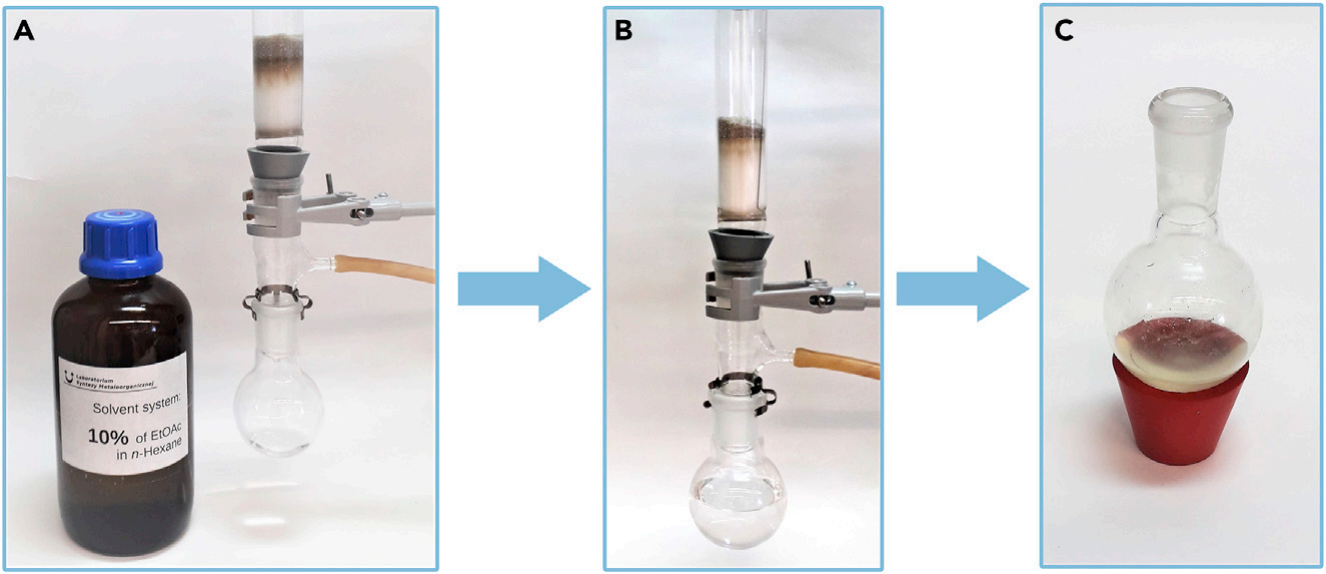
Filtration set up (A–C) (A) silica gel pad washed with 10% ethyl acetate in hexane solution (B) crude product after filtration through silica gel pad (C) crude product ready for distillation.
29. Carefully wash the product off the silica gel pad with 10%–30% solution of ethyl acetate in hexane under vacuum filtration.

**CRITICAL:** Greenish residue of the catalyst should remain on top of the silica gel pad after filtration. Any catalyst residues left in the crude product can cause its isomerization or decomposition.

**Figure 11:**
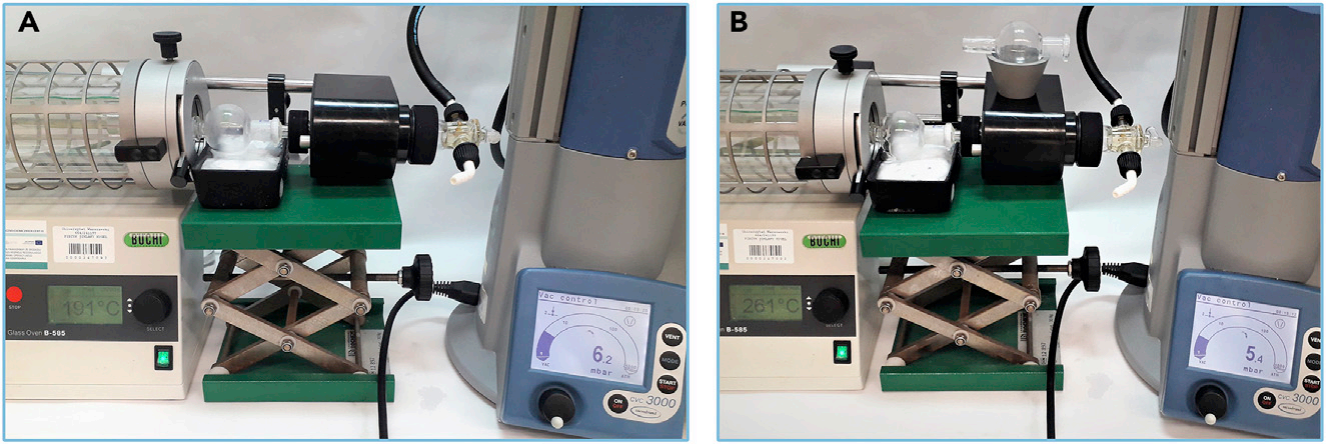
Kugel Rohr distillation (A and B) (A) beginning of the process, (B) distillation of the desired product.

30. Remove the solvent on a rotary evaporator (35°C, 130 mbar, ∼15 min).
31. Place the crude product in a small round bottom flask.
32. Purify the crude product by distillation (Figure 11):
  a. set up the parameter on Kugel Rohr distillation apparatus to distill out the unreacted starting material: 200°C, 50 rpm and 5–6 mbars on vacuum pump; troubleshooting 5.
  b. collect the first fraction in the bulb;
  c. place new bulb in the apparatus, raise the temperature to 250°C–260°C, and collect the pure product;
  d. leave a higher boiling yellow residue in the round bottom flask.

***Optional:*** Kugel Rohr apparatus can be replaced by standard laboratory glassware for distillation under vacuum.

**Figure 12:**
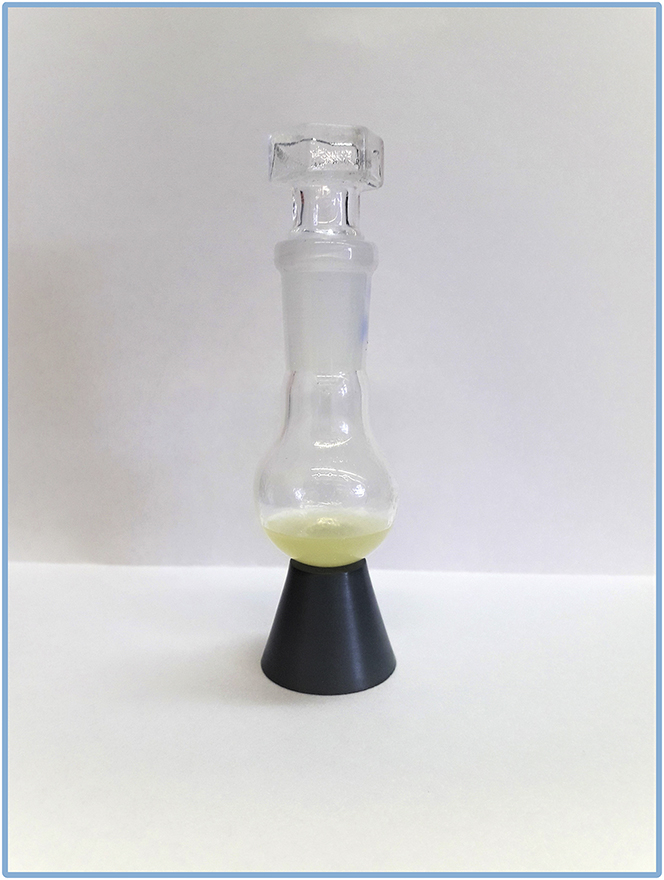
Pure 11-hydroxyundec-2-enal

33. Transfer distilled product to the fresh round bottom flask (previously tared or weighed), wash with small amount of hexane, remove the solvent, dry product under vacuum (∼3·10^−2^ mbar, at ∼20°C–25°C for 16 h) and weight out the amount of final compound (Figure 12).

## Expected outcomes

In summary, the described protocols allow for efficient synthesis and isolation of *9-chloro-2-methylnon-4-ene* and *11-hydroxyundec-2-enal* in high yields. In particular, harmful and toxic chlorinated or aromatic solvents can be replaced by an environmentally friendly solvent system: water with a small amount of ethyl acetate as a co-solvent. Accordingly, the proposed method does not require moisture and oxygen free conditions to perform the metathesis reaction, since water can serve as a convenient diluent of reactants. Moreover, a relatively low loading of ruthenium catalyst was necessary for full conversion of starting materials under mild conditions. In contrast to previously reported protocols, surfactant or specially designed catalysts are not necessary in these studies. Therefore, the developed method extends known protocols for aqueous metathesis methodology by utilizing microwave assistance and mechanical emulsification to increase the performance of the studied transformations in water. Consequently, we believe that the presented protocols can find broad applications in chemical synthesis, as the method is characterized by a low amount of waste produced, a low loading of the catalyst, and a high selectivity.

*9-chloro-2-methylnon-4-ene* (mixture of *E* and *Z* isomers) appears as a light yellow oil in 70% yield (5.76 g).

**Figure:**
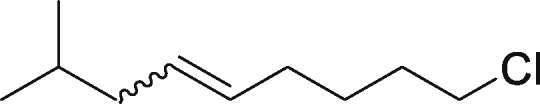

^1^H NMR (400 MHz, CDCl_3_) δ 5.52 – 5.28 (m, 2H), 3.54 (td, *J* = 6.7, 1.1 Hz, 2H), 2.09 – 1.98 (m, 2H), 1.94 – 1.83 (m, 2H), 1.83 – 1.71 (m, 2H), 1.63 – 1.45 (m, 3H), 0.89 (isomer *Z* (15%), d, *J* = 6.6 Hz, 0.9H), 0.87 (isomer *E* (85%)*,* d, *J* = 6.6 Hz, 5.1H). ^13^C NMR (101 MHz, CDCl_3_) isomer *E* δ: 130.5, 130.2, 45.0, 42.1, 36.4, 32.0, 31.8, 28.5, 26.8, 22.3; isomer *Z* δ: 130.2, 129.8, 45.0, 41.9, 36.4, 32.0, 31.7, 28.4, 26.7, 22.3. Presented analytical data are consistent with literature (Nienałtowski et al., 2020).

*11-hydroxyundec-2-enal* appears as a dense yellowish oil (solidified in the fridge) in 80% yield (1.77 g).

**Figure:**
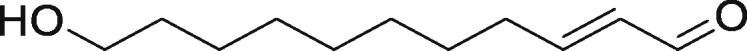

^1^H NMR (400 MHz, CDCl_3_) δ 9.50 (d, *J* = 7.9 Hz, 1H), 6.85 (dt, *J* = 15.6, 6.8 Hz, 1H), 6.11 (ddt, *J* = 15.6, 7.9, 1.5 Hz, 1H), 3.64 (t, *J* = 6.6 Hz, 2H), 2.39 – 2.24 (m, 2H), 1.60 – 1.44 (m, 4H), 1.40 – 1.28 (m, 8H). ^13^C NMR (101 MHz, CDCl_3_) δ 194.2, 159.0, 132.9, 63.0, 32.71, 32.70, 29.28, 29.25, 29.0, 27.8, 25.7. Presented analytical data are consistent with literature (Aubineau and Cossy, 2018).

## Limitations

The scope of substrates in this protocol is limited mainly to the lipophilic non-ionic compounds, as for the water soluble substrates metathesis reactions were not effective. Moreover, in such cases, isolation of the final product might be difficult.

## Troubleshooting

### Problem 1

Step 2f: During the addition of the catalyst in solid form, some of the powder may settle on the sides of the vial and reactor.

### Potential solution

Use the prepared ethyl acetate to wash the catalyst off the glass.

### Problem 2

Steps 3 and 19: Organic and aqueous layers can be difficult to separate because of stable emulsion formation.

### Potential solution

Add small amount of NaCl and shake mixture gently in the separatory funnel. This should help in layers separation. Brine can be also added.

### Problem 3

Steps 3 and 19: During extraction, both layers have an intense, dark color and it is difficult to notice the phase division.

### Potential solution

Use a flashlight to illuminate the separatory funnel on the side. Refracting rays of light will make it easier to find the interface between aqueous and organic layer.

### Problem 4

Steps 9 and 26: After the extraction step, the organic layer is not clear, but cloudy and obscure.

### Potential solution

The organic layer is not sufficiently dehydrated. The amount of drying agent depends on the amount of water remaining in the organic layer after the extraction step. When the drying agent stops clumping, there is enough of it.

### Problem 5

Steps 15a and 32a: The vacuum achieved in the distillation system is not low enough.

### Potential solution

Check that all joints are perfectly matched, and that all ground glass connections are properly lubricated with hydrocarbon based grease. If the vacuum is still not good enough despite the well-complexed distillation system, recalculate the boiling point values using the pressure-temperature nomograph.

## Resource availability

### Lead contact

Further information and requests for resources and reagents should be directed to and will be fulfilled by the lead contact, Anna Kajetanowicz (a.kajetanowicz@uw.edu.pl).

### Materials availability

This study did not generate new unique reagents. All reagents used in this study were commercially available and used without further purification.

## Data Availability

All data reported in this paper will be shared by the lead contact upon request. This paper does not report original code. Any additional information required to reanalyze the data reported in this paper is available from the lead contact upon request.
